# Supplementary figures and images for: Comparative Analysis of Transcriptional Regulation Patterns: Understanding the Gene Expression Profile in Nucleocytoviricota
Source: Pathogens. 2021 Jul 24;10(8):935. doi: 10.3390/pathogens10080935 (PMC8400408; doi:10.3390/pathogens10080935)

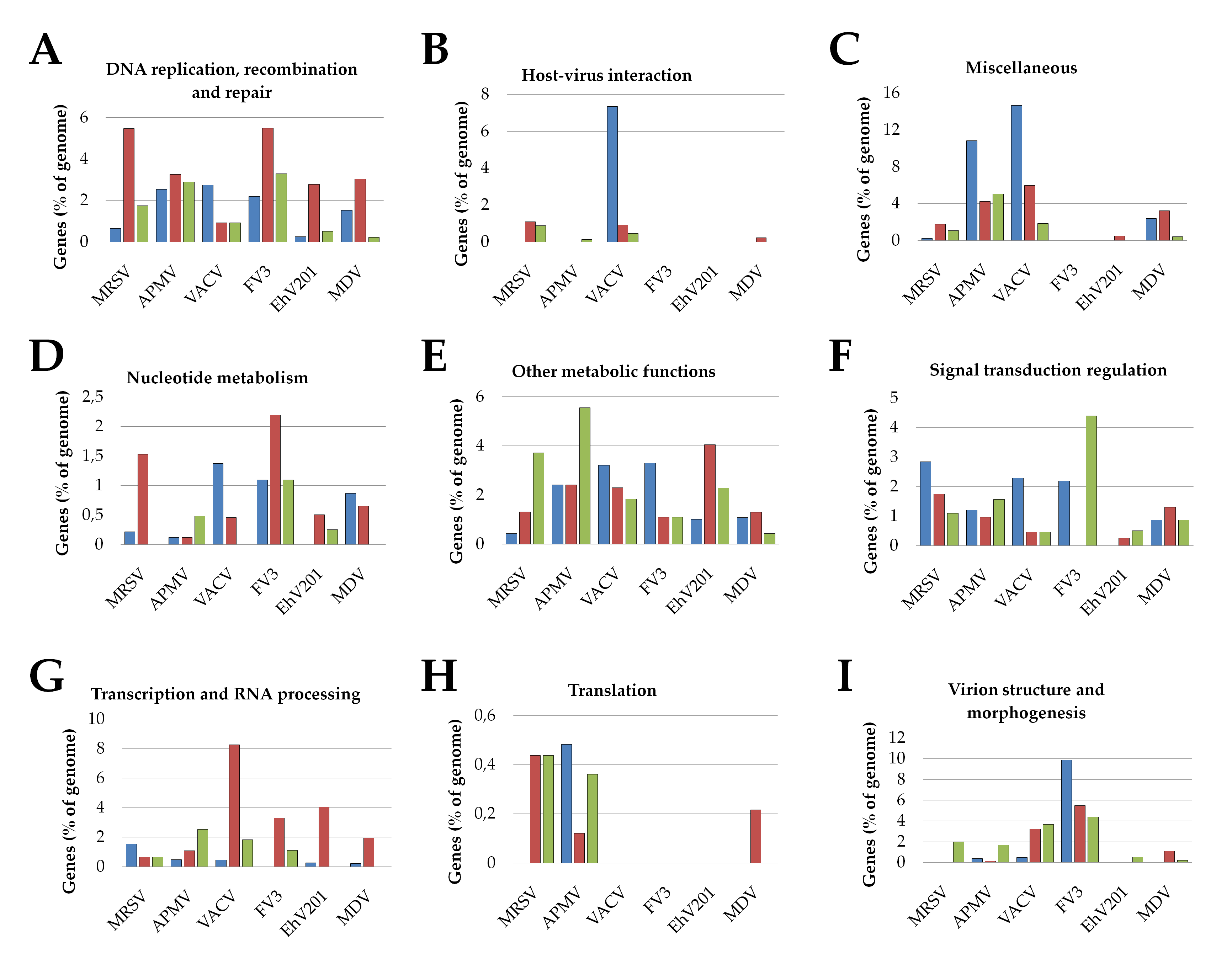

Supplement: Supplementary file 1 [file pathogens-10-00935-s001.zip › Figure S1_R1.tif]
